# Fructans As DAMPs or MAMPs: Evolutionary Prospects, Cross-Tolerance, and Multistress Resistance Potential

**DOI:** 10.3389/fpls.2016.02061

**Published:** 2017-01-11

**Authors:** Maxime Versluys, Łukasz P. Tarkowski, Wim Van den Ende

**Affiliations:** Laboratory of Molecular Plant Biology, KU LeuvenLeuven, Belgium

**Keywords:** DAMP, fructan, immunity, signaling, stress, tolerance

## Abstract

This perspective paper proposes that endogenous apoplastic fructans in fructan accumulating plants, released after stress-mediated cellular leakage, or increased by exogenous application, can act as damage-associated molecular patterns (DAMPs), priming plant innate immunity through ancient receptors and defense pathways that most probably evolved to react on microbial fructans acting as microbe-associated molecular patterns (MAMPs). The proposed model is placed in an evolutionary perspective. How this type of DAMP signaling may contribute to cross-tolerance and multistress resistance effects in plants is discussed. Besides apoplastic ATP, NAD and fructans, apoplastic polyamines, secondary metabolites, and melatonin may be considered potential players in DAMP-mediated stress signaling. It is proposed that mixtures of DAMP priming formulations hold great promise as natural and sustainable alternatives for toxic agrochemicals.

## Introduction: Damp Signaling in Plants and in Animals

Throughout their lifecycle, plants are prone to different sorts of stresses, many of which cause cellular rupture. In case of biotic stress, the recognition of molecular patterns from microbes (microbe-associated molecular patterns, MAMPs) or herbivores (herbivore-associated molecular patterns, HAMPs) is well-known, especially for MAMPs. However, more recently the importance of damaged-self recognition has come to light. The manuscript by Duran-Flores and Heil (2016) highlights the significance of DAMPs (damage-associated molecular patterns) in response to cellular disruption. The role of DAMPs in animals has been proposed as the so-called danger model (Matzinger, 1994). Different molecules were proposed as DAMPs, including extracellular ATP and mitochondrial DNA (Krysko et al., 2011; Crišan et al., 2016). Recently, Martin (2016) proposed members of the IL-1 (interleukin 1) family of cytokines as the canonical DAMPs in animals, indicating how well-studied molecular structures can have a yet unknown function as DAMPs. However, research on DAMP signaling in plants is still in its infancy, although DAMP-mediated signaling was proposed as one of the central players in plant defense priming (Martinez-Medina et al., 2016). As of late, Heil (2012), Heil and Land (2014), Heil et al. (2016) have put the debate on DAMPs into the spotlight by discussing evolutionary benefits as well as features ascribable to DAMPs.

## Sugars as Damps: the Case of Plant Fructans

In their latest manuscript, Duran-Flores and Heil (2016) include sucrose (Suc), a central transport and signaling sugar in plants (Smeekens and Hellmann, 2014) for the first time as a DAMP in their scheme, associated with plant defense responses. The recently launched “sweet immunity” concept attempts to explain the role of (sweet) small sugars, and by extension, less sweet carbohydrates with a higher degree of polymerization (DP) in plant innate immunity responses. Considering biotic stress responses, small metabolic sugars are not only a possible food source for the pathogen, but can act as signaling molecules to induce plant defense response (Bolouri Moghaddam and Van den Ende, 2012, 2013), with a central role for the SnRK1 energy sensor (Van den Ende and El-Esawe, 2014; Hulsmans et al., 2016).

Fructans are polysaccharides synthesized in the vacuole of 15% of flowering species (Van den Ende et al., 2004). Fructose moieties are added to Suc by various fructosyltransferases. Different types of fructans are found in plants, depending on the linkage type and branching, including inulins, levans, graminans, and neokestose-type inulins and levans as well as complex, mixed-type fructans from *Agave* sp., the agavins (Mancilla-Margalli and López, 2006; Valluru and Van den Ende, 2008; Van den Ende, 2013).

Here, we propose a possible role of fructans as DAMPs in fructan accumulating plants. Livingston and Henson (1998) detected an increase in apoplastic fructan content after subzero acclimation in oat (*Avena sativa*). Their presence in the apoplastic environment after a stress event may suggest a possible role as DAMPs. Recently, it was found that short inulin-type fructans (fructooligosaccharides, FOS) from *Arctium lappa* or burdock (burdock fructooligosaccharides, BFO) prime plant defenses in different pathosystems. (Wang et al., 2009; Zhang et al., 2009; Sun et al., 2013). Priming, a process believed to occur at the expense of minimal amounts of ATP, brings plants in a “ready-to-go” status, preparing them for a faster and stronger response to future (a)biotic stresses (Conrath, 2015).

## Bacterial Fructans Acting as Mamps in Plants?

Although, the above-mentioned plant fructan priming function may involve DAMP signaling in fructan accumulating plants, it is important to realize that fructans are also present in bacteria and fungi. While levan-type fructans are widespread in microorganisms, inulin-type fructans are only found in certain genera of gram-positive bacteria (Toksoy et al., 2016 and references therein). Genera such as *Lactobacillus* and *Streptococcus* produce levans extracellularly. In *Lactobacillus*, production of either levans or inulins has been found in related strains (Ozimek et al., 2006; Anwar et al., 2010). Fructans increase virulence of pathogenic species through mechanisms such as biofilm formation and Ca^2+^-chelation to suppress host defenses, as reported in *Erwinia amylovora* (Koczan et al., 2009; Ordax et al., 2010; Ichinose et al., 2013).

Importantly, the DP of these bacterial fructans is much higher than those occurring in plant fructans (Toksoy et al., 2016). Thus, bacterial fructans are likely immobile within the plant cell wall. More likely, FOS derived from their (partial) hydrolysis by plant apoplastic fructan exohydrolases (FEHs) (Van den Ende et al., 2004) may readily diffuse through the plant apoplast to trigger potential defense-related receptors present in the plant plasma membrane (PM). As such, bacterial FOS may be recognized as MAMPs in plants, sensed by so far unidentified receptors.

## A Possible Comparison With Fructan-Mediated Immune Signaling in Animals?

Referring to the situation in animals and humans, inulin-type fructans, besides indirectly activating microorganisms in the colon, are believed to be directly recognized by host receptors in the gut system, such as toll-like receptors 2 and 4 (TLR2 and TLR4) (Vogt et al., 2013; Peshev and Van den Ende, 2014; Franco-Robles and López, 2015). This primes innate immunity and contributes to better health. Fructans interact with a lower affinity with TLR2 and TLR4 as compared to bacterial lipo-oligosaccharides (LPS) (Takeuchi et al., 1999).

So far, most research is focused on inulin-type fructans derived from chicory (*Cichorium intybus*), but other types of plant fructans such as agavins (*Agave tequilana*, López-Velázquez et al., 2015) and graminans (cereals, Verspreet et al., 2015) are under study. Dietary fructans are degraded by fructan-degrading enzymes from microbes in the colon, since animals lack fructan-degrading enzymes (Capitán-Cañadas et al., 2014; Peshev and Van den Ende, 2014). Dietary supplements of bacterial levans are also known to improve growth and defense responses in different animal species (Li and Kim, 2013; Huang et al., 2015). Anti-tumor and immunomodulatory effects have been ascribed to some bacterial levans (Yoo et al., 2004; Xu et al., 2006). Since animals and humans lack enzymes that can biosynthesize fructans, fructans cannot act as DAMPs. It can be speculated that TLR2 and 4 may both recognize bacterial and plant-derived fructans, although this remains to be proven. Bacterial fructans can be considered as MAMPs in this case. Since TLR2 and TLR4 homologs are absent in plant genomes, it seems that other, so far unidentified fructan receptors were recruited in the evolutionary lineage leading to plants.

## Fructan: Mamps, Damps, or Both?

**FIGURE 1:**
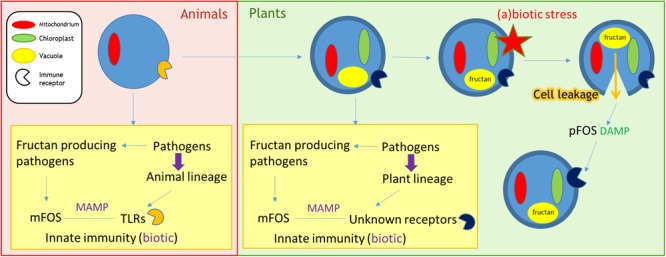
**Role of fructans as MAMPs/DAMPs.** Pathogens exert evolutionary pressure on host animals/plants. In case of fructan producing pathogens, these mFOS can function as a virulence factor. However, during biotic interactions, they may be recognized as MAMPs by the host. Selection will favor hosts that recognize mFOS through specific receptors that induce immune responses. In animals, microbial fructooligosaccharides (mFOS) from fructan producing pathogens are recognized by TLRs, thereby activating innate immune responses. A similar mechanism may be present in plants, where mFOS from fructan producing phytopathogens bind to currently unknown receptors to induce defense responses. Besides a MAMP recognition mechanism, fructans may also be involved in damaged-self recognition in certain plant species. 15% of flowering species synthesize and store fructans (plant fructooligosaccharides or pFOS) in the vacuole. In these plants, pFOS can be perceived as DAMPs by the unknown fructan receptors involved in MAMP recognition. Cellular rupture after (a)biotic stress will leak the stored pFOS into the apoplastic environment, triggering neighboring cells to upregulate immune responses after pFOS recognition by these receptors. As such, mild abiotic stresses may enhance disease resistance against future pathogen attack. DAMP, damage-associated molecular pattern; MAMP, microbe-associated molecular pattern; mFOS, microbial fructooligosaccharides; pFOS, plant fructooligosaccharides; TLR, toll-like receptor.

Both MAMPs and DAMPs are currently accepted as immune response inducers (Cook et al., 2015). So are fructans MAMPs, DAMPs, or both? The model that we propose suggests both, with the speculation that an evolutionary event resulted in a switch of fructan perception from MAMP to DAMP in fructan accumulating plants (**Figure 1**). In animals, the recognition of microbial fructans by PM-localized TLRs has been documented (see above), thus activating innate immunity. The possibility exists that the same holds true in plants, where shorter microbial fructooligosaccharides (mFOS) diffuse through the cell wall acting as MAMPs to activate immune responses. However, a receptor for fructans has not been described so far. Fructans are stored in the vacuole of fructan-accumulating plants. Within the damaged-self context, lysed cells releasing their fructan content may lead to partial fructan degradation in the apoplast. The derived plant fructooligosaccharides (pFOS) are expected to be more mobile, diffusing to neighboring cells where they are possibly sensed by ancient receptors (putatively localized in the PM), that are actually involved in fructan MAMP recognition (**Figure 1**).

## Fructans, Damp Signaling, and Cross-Tolerance

Within such framework (**Figure 1**), mild abiotic stresses may positively influence disease tolerance. If only some cells are damaged, the released mixture of DAMPs (including fructans and other compounds, see below) can prime the surrounding cells, hence priming their native immune system, thus increasing tolerance to a future pathogenic attack. The process in which resistance toward a specific stress is achieved through exposure to another (milder form of) particular stress is known as cross-tolerance. After exposure to a first stress stimulus, the plant is in a primed or hardened state, allowing it to respond to future stresses in a faster and stronger way (Rejeb et al., 2014; Savvides et al., 2016). Some examples can be found in the literature where abiotic stress exposure leads to increased biotic stress resistance. In *Arabidopsis thaliana*, ozone exposure triggers an induced resistance, associated with the expression of numerous defense-related genes, while drought stress increases resistance to pathogen infection through ROS in *Nicotiana benthamiana* (Sharma et al., 1996; Ramegowda et al., 2013). Thus, the damaged-self hypothesis and sweet immunity model predict an induction of plant defenses under mild stress conditions. During severe drought, however, Ramegowda and Senthil-Kumar (2015) proposed that massive cellular leakage of nutrients in the apoplast promotes infection. One possible scenario is that promotion of microbial growth by sugars in excess (or any and other nutrients) overrules signaling effects that could lead to increased plant protection.

In particular, the effects of cold stress on disease resistance have been well-described. Gene expression assays in *Vitis amurensis* indicate an upregulation of genes involved in innate immune system responses after cold acclimation (Wu et al., 2014; Moyer et al., 2015). Most research has focused on cold hardening and subzero acclimation of fructan accumulating cereals. In wheat (*Triticum aestivum*), fructan accumulates in response to low temperatures (Meguro-Maoka and Yoshida, 2016) through an increase in enzymatic activity of enzymes involved in fructan biosynthesis (Kawakami and Yoshida, 2002, 2005). Interestingly, the DP of these fructans increases from autumn to winter. Subzero acclimated plants have high contents of graminan-type fructans, characterized by branched structures (Yoshida and Kawakami, 2013). This process is most likely associated with increased apoplastic fructan levels, as observed in subzero acclimated oat (Livingston et al., 1993; Livingston and Henson, 1998), and correlates well with increased tolerance against snow mold infections. Snow molds are fungi with the ability to infect plants under snow at around 0°C (Gaudet and Laroche, 1997). Resistant wheat cultivars display higher fructan levels toward early winter and lower fructan degradation under snow in comparison to susceptible cultivars (Yoshida et al., 1998; Iriki et al., 2005; Nishio et al., 2008; Kawakami and Yoshida, 2012).

## Damp Mixtures for Multistress Resistance

Designating fructans as damaged-self signaling molecules in fructan accumulating plants may not be so far-fetched. If the receptors involved are evolutionarily conserved, fructan accumulating plants may sense endogenous fructans as DAMPs, and bacterial fructans as MAMPs. A signaling function for less common sugars, like we propose here for fructans, has been described before. In gentians, gentiobiose appears to be involved in signaling budbreak in overwintering buds (Takahashi et al., 2014). Nevertheless, although we propose fructans as DAMPs in fructan accumulating plants, we must keep in mind that these are only one of many (possible) DAMPs that are released into the apoplast after cellular rupture. Thus, their contribution to priming innate immunity may be limited. Likely, a mixture of DAMPs rather than one released compound will induce an efficient priming.

What are other powerful DAMPs putatively involved in defense priming? Extracellular ATP is a central signaling molecule in plant stress responses, sensed by the PM receptor DORN1 (Cao et al., 2014). Similarly, extracellular NAD was proposed to act as a DAMP in *Arabidopsis* (Zhang and Mou, 2009; Pétriacq et al., 2016). Polyamines (PAs) such as spermine, spermidine, and putrescine are generally found in plant cells (Hussain et al., 2011; Minocha et al., 2014; Pál et al., 2015) and exogenous application revealed good priming potential (Li et al., 2015; Nahar et al., 2015), suggesting that they may be considered to be candidate DAMPs as well. Accordingly, mild salt stress increases apoplastic PA levels (Moschou et al., 2008). It is well-known that apoplastic PAs play important roles in plant-pathogen interactions, leading to significant changes in host susceptibility to different kinds of pathogens (Marina et al., 2008). Although, these have been explained by hydrogen peroxide-mediated signaling originating from PA oxidation in the apoplast, the view that apoplastic PAs may directly trigger immune receptors in the PM involved in DAMP signaling should be reconsidered.

Similarly, secondary metabolites such as naringenin, quercetin, and rutin may be considered as candidate DAMPs as well. Indeed, exogenous naringenin treatment led to increased drought tolerance (Pourcel et al., 2013), while quercetin and rutin priming led to increased resistance against bacterial pathogens in *Arabidopsis* (Jia et al., 2010; Yang et al., 2016). A screening of an array of mutants revealed that flavonoids are determinants of freezing tolerance and cold acclimation in *Arabidopsis* (Schulz et al., 2016). Taken all together, this suggests that some secondary metabolites can be used as signaling compounds to counteract both abiotic and biotic stresses.

Also melatonin, an indoleamine, has a strong priming potential when applied exogenously (Shi et al., 2014). Furthermore, a link between melatonin and sugar metabolism and signaling has been suggested in the context of biotic stress (Zhao et al., 2015). Recently, Jiao et al. (2016) isolated endophytic bacterial strains that live in the plant apoplast and secrete melatonin. Colonization by one such strain protected plants from adverse effects of salt or drought stress through upregulation of intracellular melatonin biosynthesis in the host plant. Thus, apoplastic melatonin levels somehow interact with intracellular melatonin levels, and such mechanisms may be tightly interlinked to damage-self recognition processes originating in the apoplastic continuum under various stresses.

Recently, Bruce et al. (2016) and Savvides et al. (2016) discuss the possibilities of chemical priming on multistress resistance, a popular topic in current research focusing on developing natural and sustainable alternatives for toxic agrochemicals. It is likely, that mixtures of priming agents can lead to synergistic effects and increased multistress tolerance by reflecting to what occurs when a complex mixture of intracellular metabolites is released in the apoplast after cellular rupture. Therefore, future research should focus on the priming efficacy of cocktails of the above-mentioned compounds in combination with different types of fructans from plant and microbial origin.

## Fructans and Glucans: a Comparison

The view that fructans act as MAMPs and/or DAMPs may also hold true for other classes of polysaccharides such as β-glucans, containing glucose- instead of fructose moieties. β-1,3- and β-1,6-glucans represent a significant part of fungal cell walls (Dalonso et al., 2015). β-1,3- and β-1,4-glucans are also present in the cell walls of most plants of the Poaceae and in *Equisetum*, as well as in bryophytes. The highest abundance is found in cereals (Gibeaut et al., 2005; Burton and Fincher, 2009). The recognition of fungal β-glucans by the Dectin-1 receptor in animals was investigated thoroughly. This receptor has been discovered by Brown and Gordon (2001) and downstream responses have been characterized (Brown, 2006; Plato et al., 2015). Recently, Sahasrabudhe et al. (2016a) reported that pre-digestion of oat β-glucan with an endo-glucanase enhances the activation state of the Dectin-1 receptor in human dendritic cells. This observation fits well with the idea that shorter DP glucans, as well as fructans, may be considered as priming agents boosting native immunity both in animals and in plants.

In plants, only a few examples of β-glucan recognition are present. In soybean (*Glycine max*) it has been shown that a β-glucan binding protein can recognize β-glucans of the oomycete *Phytophthora megasperma* (Fliegmann et al., 2004). In a recent manuscript, analysis of key enzymes in β-1,6-glucan biosynthesis in *Colletotrichum graminicola* revealed a downregulation of this biosynthesis pathway in biotrophic hyphae in order to attenuate immune responses of the host (Oliveira-Garcia and Deising, 2016). Besides their possible function as MAMPs, these β-glucans could also function as DAMPs in cereals.

Other examples include arabinoxylans, which increase phagocytosis in macrophages and induce anti-inflammatory effects (Ghoneum and Matsuura, 2004; Kang et al., 2016). Accordingly, arabinoxylan activates Dectin-1 and modulates particulate β-glucan-induced Dectin-1 activation (Sahasrabudhe et al., 2016b).

## Conclusion

While research on DAMP signaling in plants is still in an early phase, this perspective paper proposes possibilities for new and inventive experiments. The potential role of microbial fructans as MAMP in plants and plant fructans as DAMP in fructan accumulating plants is explained and compared to the case of glucans. While microbial fructan perception in animals has been characterized, the situation in plants is still unclear and identification of a fructan receptor requires further investigation. We propose that through such evolutionary ancient mechanism, plant-derived fructans, as potential DAMPs, may prime the immune system of fructan accumulating plants. Within this framework, the role of DAMP signaling in multistress resistance is discussed and other potential DAMPs, such as PAs and secondary metabolites, may be important players in (a)biotic stress tolerance as well. The potential use of mixtures of DAMPs for priming requires further investigation and may provide promising alternatives for toxic agrochemicals.

## Author Contributions

MV and WVdE defined the perspective. MV wrote the first draft, input was provided by ŁPT and WVdE.

## Conflict of Interest Statement

The authors declare that the research was conducted in the absence of any commercial or financial relationships that could be construed as a potential conflict of interest.

## Abbreviations

ATP: adenosine triphosphate
BFO: burdock fructooligosaccharides
DAMP: damage-associated molecular pattern
DORN1: does not respond to nucleotides 1
DP: degree of polymerization
FEH: fructan exohydrolase
FOS: fructooligosaccharides
HAMP: herbivore-associated molecular pattern
IL-1: interleukin 1
LPS: lipo-oligosaccharides
MAMP: microbe-associated molecular pattern
mFOS: microbial fructooligosaccharides
NAD: nicotinamide adenine dinucleotide
PA: polyamine
pFOS: plant fructooligosaccharides
PM: plasma membrane
ROS: reactive oxygen species
SNF1: sucrose non-fermenting 1
SnRK1: SNF1-related kinase 1
Suc: sucrose
TLR: toll-like receptor
